# Micelle-Formulated Juglone Effectively Targets Pancreatic Cancer and Remodels the Tumor Microenvironment

**DOI:** 10.3390/pharmaceutics15122651

**Published:** 2023-11-21

**Authors:** Vidhi M. Shah, Syed Rizvi, Alexander Smith, Motoyuki Tsuda, Madeline Krieger, Carl Pelz, Kevin MacPherson, Jenny Eng, Koei Chin, Michael W. Munks, Colin J. Daniel, Adel Al-Fatease, Galip Gürkan Yardimci, Ellen M. Langer, Jonathan R. Brody, Brett C. Sheppard, Adam WG. Alani, Rosalie C. Sears

**Affiliations:** 1 Brenden-Colson Center for Pancreatic Care, Oregon Health and Science University, 3181 Southwest Sam Jackson Park Road, Portland, OR 97239, USA; shahv@ohsu.edu (V.M.S.);; 2 Department of Pharmaceutical Sciences, College of Pharmacy, Oregon State University, 2730 South Moody Avenue, Portland, OR 97201, USA; 3 Department of Molecular and Medical Genetics, Oregon Health and Science University, 3181 Southwest Sam Jackson Park Road, Portland, OR 97239, USA; 4 Cancer Early Detection Advanced Research Center, School of Medicine, Oregon Health and Science University, Portland, OR 97239, USA; 5 Knight Cancer Institute, Oregon Health & Science University, Portland, OR 97201, USA; 6 Department of Biomedical Engineering, Oregon Health and Science University, 3181 Southwest Sam Jackson Park Road, Portland, OR 97239, USA; 7 Department of Pharmaceutics, College of Pharmacy, King Khalid University, Guraiger, Abha 62529, Saudi Arabia; 8 Department of Surgery, Oregon Health and Science University, 3181 Southwest Sam Jackson Park Road, Portland, OR 97239, USA

**Keywords:** juglone, drug delivery, pancreatic cancer, micelles, nanotechnology

## Abstract

Pancreatic cancer remains a formidable challenge due to limited treatment options and its aggressive nature. In recent years, the naturally occurring anticancer compound juglone has emerged as a potential therapeutic candidate, showing promising results in inhibiting tumor growth and inducing cancer cell apoptosis. However, concerns over its toxicity have hampered juglone’s clinical application. To address this issue, we have explored the use of polymeric micelles as a delivery system for juglone in pancreatic cancer treatment. These micelles, formulated using Poloxamer 407 and D-α-Tocopherol polyethylene glycol 1000 succinate, offer an innovative solution to enhance juglone’s therapeutic potential while minimizing toxicity. In-vitro studies have demonstrated that micelle-formulated juglone (JM) effectively decreases proliferation and migration and increases apoptosis in pancreatic cancer cell lines. Importantly, in-vivo, JM exhibited no toxicity, allowing for increased dosing frequency compared to free drug administration. In mice, JM significantly reduced tumor growth in subcutaneous xenograft and orthotopic pancreatic cancer models. Beyond its direct antitumor effects, JM treatment also influenced the tumor microenvironment. In immunocompetent mice, JM increased immune cell infiltration and decreased stromal deposition and activation markers, suggesting an immunomodulatory role. To understand JM’s mechanism of action, we conducted RNA sequencing and subsequent differential expression analysis on tumors that were treated with JM. The administration of JM treatment reduced the expression levels of the oncogenic protein MYC, thereby emphasizing its potential as a focused, therapeutic intervention. In conclusion, the polymeric micelles-mediated delivery of juglone holds excellent promise in pancreatic cancer therapy. This approach offers improved drug delivery, reduced toxicity, and enhanced therapeutic efficacy.

## 1. Introduction

Pancreatic cancer is a devasting disease with a current five-year survival rate of just 12%. The American Cancer Society estimates that about 79% of the 64,050 people diagnosed with pancreatic cancer in 2023 will die (*Cancer Facts & Figures 2023*, American Cancer Society, Atlanta, GA, USA, 2023). Moreover, pancreatic ductal adenocarcinoma (PDAC) accounts for over 90% of pancreatic cancer cases. The challenge with PDAC is the lack of symptoms in the early disease, thus obfuscating surgery, the only potentially curative treatment option. While resection and standard-of-care chemotherapy combinations such as gemcitabine plus nab-paclitaxel and FOLFIRINOX have managed to benefit patients with early-stage pancreatic cancer, chemotherapy has limited and only short-term activity for patients in the late stages of the disease [1]. While immunotherapies have dramatically affected some solid tumors, such as lung and melanoma, such success has not manifested in PDAC, which is commonly considered nonimmunogenic and exhibits an immune-suppressive microenvironment [2,3].

The aggressive and therapeutic-resistant nature of PDAC is thought to exist both at the genetic and cellular levels, where it is challenging to target oncogenes, and where tumor cell heterogeneity predominates in the disease. While new KRAS inhibitors are exciting in this disease where mutant KRAS is a near-universal driver, resistance is likely to develop rapidly, based on initial trials [4]. In addition, cellular plasticity and the presence of cancer stem-like cells contribute to therapeutic resistance. Current therapies do not adequately target cancer stem cells, which make up approximately 1% of pancreatic cancer cells and have been shown to contribute to tumor development and treatment resistance [5]. In addition, the high metastatic potential of PDAC further reduces the efficacy of current treatments. These factors suggest that therapeutic interventions targeting individual pathways will eventually result in drug resistance, limiting our clinical treatment options for pancreatic disease. This scenario indicates that using natural compounds, which typically target multiple molecular pathways and manifest various mechanisms of action, may result in higher PDAC survival rates. Experiments on different cancer types have demonstrated the anticancer efficacy of natural compounds [6,7,8]. In the last five years, approximately 70 natural products have been studied and reported in relation to pancreatic cancer [9], seven of which have been evaluated in clinical trials for treating pancreatic cancer, of which five were conducted in the United States. Four of the seven clinical trials were completed in Phase 2, one was in Phase 3, and one was still enrolling participants for Phase 3. All completed clinical trials showed a favorable response and increased survival compared to the control group [9].

Juglone (5-hydroxy-1,4-naphthoquinone) is a natural 1,4-naphthoquinone found in walnut trees. Juglone has been reported to possess cytotoxic properties against human gastric, leukemia, cervical, colon, and pancreatic cancer cell lines confirmed through in vitro assays [10,11,12,13,14,15,16,17]. The studies have reported that juglone exhibits cytotoxic properties by inducing apoptosis through mitochondria-dependent pathways [10,12,15,16]. Although the antitumor activity of juglone is quite robust, in vivo efficacy studies on it are limited due to its poor water solubility (0.04 mg/mL). Secondly, such in-vivo studies were conducted for a short time < 14 days and dosed every two days, perhaps due to the compound’s toxicity. In the present study, nano-scaled polymeric micelles were designed for the delivery of juglone to pancreatic tumors in vivo.

Polymeric micelles have gained extensive attention as a delivery system for poorly water-soluble drugs as they increase solubility and promote accumulation and retention into tumor tissue via enhanced permeability and retention effects [18,19]. Micelles have a diameter typically ranging from 10 to 200 nm [18,19,20]. They form spontaneously due to the aggregation of amphiphilic molecules, in which the hydrophobic chain of the polymers forms the core, and the hydrophilic ends form the structural shell [18,19]. Pluronics are block copolymers, which are widely used as micellar carriers [21]. Pluronic comprises hydrophilic polyethylene oxide (PEO) and hydrophobic poly propylene oxide (PPO) segments arranged in a basic tri-block structure: PEO–PPO–PEO [22]. Pluronic F127(F127) (PEO100-PPO69-PEO100) possesses an extended PEO block that improves the encapsulation efficacy, stability, and circulation time of drugs [21]. The Food and Drug Administration acknowledges F127 as a safe pharmaceutical adjuvant, and its biocompatibility contributes to its widespread application as a nanodrug delivery polymer [22].

F127 generates micelles at a low critical micelle concentration (CMC) of 0.0031% *w*/*w* and does not inhibit P-glycoprotein, a multidrug resistance (MDR) protein and an essential efflux drug transporter protein [23,24]. As a result, F127 micelles frequently have a low loading efficiency and a larger particle size. Published work [25] indicates that each of these characteristics can potentially limit both the dose and the ability of micelles to cross biological barriers. Micelles containing an additional lipophilic copolymer may provide a superior platform by increasing loading and decreasing particle size to deliver hydrophobic drugs, such as juglone.

D-α-Tocopherol polyethylene glycol 1000 succinate (TPGS) is a non-ionic, water-soluble vitamin E derivative formed by conjugating vitamin E succinate with polyethylene glycol [26]. It has been authorized by the Food and Drug Administration as a safe pharmaceutical adjuvant and is used in various drug formulations [26]. In addition, TPGS has other advantages that make it an ideal nanocarrier for poorly soluble drug delivery. First, unlike F127, TPGS enhances the cellular uptake of the drugs [27] by reversing MDR and inhibiting a P-glycoprotein-mediated efflux of drugs [28]. Second, TPGS has been used as a drug carrier to form prodrugs for chemotherapeutical drugs, such as doxorubicin, paclitaxel, and gemcitabine, to promote drug delivery to tumors and to reverse MDR in cancer therapy. Third, TPGS possesses antioxidant activity [29], which can protect pharmaceuticals from oxidative degradation during storage, thereby enhancing formulation stability. However, the CMC of TPGS is relatively high (0.02%, *w*/*w*), which may accelerate the dissociation of micelles in plasma. Therefore, we formulated mixed micelles with TPGS and F127 to deliver juglone as a nanoformulation suitable for in vivo evaluation, circumventing its poor water solubility and potentially enhancing its stability and circulation time for treating pancreatic cancer.

In this work, we prepared juglone micelles (JMs) using a solvent-casting method. The micelles were characterized for particle size distribution, morphological observation, zeta potential, and encapsulation efficiency. Then, we evaluated JM for its in vitro effects on pancreatic cancer cell lines and in vivo effects on tumor growth in both immunocompromised and immunocompetent pancreatic tumor mouse models. Bulk RNA sequencing was performed on pancreatic tumors treated with the JM to understand changes to the global transcriptomics following juglone delivery. Taking advantage of the therapeutic properties of juglone, we developed a polymeric micelle formulation that induces a significant reduction in tumor growth in both immunocompetent and immunocompromised mice, thus making the evaluation and therapeutic application of juglone feasible in in vivo pancreatic tumor models.

## 2. Materials and Methods

### 2.1. Materials

Juglone (Fischer Scientific, Waltham, MA, USA) (CAS 48139-0), Pluronics F-127 (Letco Medical, Decatur, AL, USA) (CAS 9003-11-6), D-alpha-Tocopherol polyethylene glycol 1000 succinate (TPGS), BioXtra, and water-soluble vitamin E conjugate (Sigma Aldrich, Burlington, MA, USA) (CAS 9002-96-4) were used for the in vitro micelle preparation.

### 2.2. Cell Culture

Human pancreatic ductal adenocarcinoma cell lines: Panc-1 and MiaPaCa-2 cells were purchased from ATCC and were cultured in Dulbecco’s Modified Eagle Medium (DMEM) (HyClone), supplemented with 10% (*v*/*v*) fetal bovine serum (FBS) (Gibco, Waltham, MA, USA) and 1% (*v*/*v*) penicillin-streptomycin (HyClone, Logan, UT, USA). All cells were routinely maintained at 37 °C under 5% CO_2_ in a humidified incubator.

### 2.3. Preparation of Juglone Micelles (JMs) and Characterization

Polymeric micelle formulation of juglone was prepared using the solvent casting method using Pluronic F127 and TPGS. Briefly, 45 mg of Pluronic F-127 and 11 mg of TPGS were weighed and mixed in 1 mL of 100% ethanol. Approximately 2.8 mg of juglone from a stock solution of 8 mg/mL was spiked into the polymer mix, vortexed gently, and subjected to solvent evaporation using a rotary evaporator to form a thin film. Once the formed film cooled at room temperature, 1 mL of saline was added, and a round-bottom flask was allowed to rotate in the water bath to ensure complete hydration of the film. The micelles were spun down at 6000 rpm for 6 min at room temperature. The supernatant was filtered and collected using a 0.2 µm syringe filter. Drug loading and encapsulation efficiency were quantified using reverse-phase, high-performance liquid chromatography (RP-HPLC), (Shimadzu, Canby, OR, USA). The analysis was performed using a Zorbax C18 column (4.6 × 75 mm, 3.5 μm) (Agilent Technologies, Santa Clara, CA, USA) in an isocratic mode with acetonitrile/water (50/50) containing 0.1% phosphoric acid and 1% methanol at a flow rate of 1.5 mL/min, and an injection volume of 10 μL. Column temperature was maintained at 40 °C with a run time of 3 min. The juglone peak is monitored at 250 nm and has a retention time of 1.5 min. The micelles were dissolved in ethanol to extract the encapsulated juglone for quantification. The encapsulation efficiency (EE%) was calculated using the formula below: %EE = ((drug amount entrapped in the micelles/total amount of drug added) × 100%). The size distribution of micelles was measured using a dynamic light-scattering instrument (Malvern Zetasizer Nano ZS; Malvern Instruments, Worcestershire, UK). The data are presented as %EE, Zave ± SD, and PDI ± SD for four replicates.

The morphology of the micelles was observed under a transmission electron microscope (TEM; Philips CM 120, Amsterdam, the Netherlands). A drop of micellar solution was placed on a copper grid, covered with a carbon film, and stained using uranyl acetate solution (2%, *w*/*v*). After dyeing at room temperature, the sample was observed under the TEM.

To assess the physical stability of the juglone-loaded F127/TPGS mixed micelles, we incubated the micelles at room temperature for 24 h. At 24 h, the micelles were centrifuged at 6000 rpm for 6 min at room temperature. The supernatant was collected and filtered through a 0.22 μm filter membrane, followed by dilution with ethanol. The diluted supernatant was used to measure the concentration of juglone retained in micelles using high-performance liquid chromatography (Cary Elipse, Agilent, Santa Clara, CA, USA).

### 2.4. In Vitro Cell Viability

Panc-1 and MiaPaCa-2 cells were seeded on 96-well plates at a density of 2000 cells/well and allowed to grow as a monolayer for 24 h before drug treatment. Cells were then treated with blank micelle particles, free juglone, and JM particles for 48 h. Untreated cells served as a control. At the start and end of the 48 h period, cell viability was measured using a 3-(4,5-Dimethylthiazol-2-yl)-5-(3-carboxymethoxyphenyl)-2-(4-sulfophenyl)-2H-tetrazolium (MTS) assay. Briefly, 10 μL of MTS (10 mg/mL) was added to 96-well plates and cultured for an additional 1 h. The absorbance value (OD) was determined using a BioSpa Cytation5 (BioTek) reader (Agilent Technologies, Santa Clara, CA, USA) at 490 nm. The following equation calculated the percentage of cell viability:

Percentage of cell viability = (OD_sample_ at 72 h/OD_cells_ at day 0) × 100

The half maximal inhibitory concentration (IC50) of free juglone and JM particles were further calculated from their inhibition rates on cell viability by using GraphPad Prism version 9.1 (La Jolla, CA, USA).

### 2.5. Cell Migration Assay

MiaPaca-2 cells (2 × 10^5^ cells/well) were seeded in 24-well plates and allowed to adhere overnight. At 80–90% confluence, a “reference line” was scratched at the bottom of the plate using a sterile 200 μL pipette tip. After being washed with phosphate buffer saline (PBS) thrice, cells were further incubated with juglone and JMs (0.6, 1.2, 2.4, and 4.8 μM) or a vehicle (DMSO). Images of cells migrating across the reference line were taken in different fields with an Evos after treatment at 0 and 24 h, respectively. The wound closure was calculated by measuring the wound area using ImageJ software; the % area at 24 h was normalized to the area at 0 h and plotted as a function of both juglone and JM concentration.

### 2.6. Apoptosis Assay

Apoptosis was evaluated using the Annexin V/PI Kit (BD Biosciences, San Jose, CA, USA). Exponentially growing cells were seeded on 6-well plates and then treated with juglone and JM (0.6, 1.2, 2.4, 4.8, and 9.6 μM) or DMSO (vehicle control, final concentration of 0.1%) for 24 h. The treated cells were harvested and stained with Annexin V and propidium iodide (PI). Flow cytometry was conducted using Cytek Aurora (Cytek Biosciences, Fremont, CA, USA).

### 2.7. Mouse Models

Six-to-eight-week-old male and female nude mice and C57BL/6 were purchased from Jackson Laboratory (Farmington, CT, USA) and housed in a pathogen-free barrier room. All animal studies complied with Oregon Health & Science University (OHSU) animal use guidelines and were approved by the OHSU Institutional Animal Care and Use Committee (protocol number TR1_IP00001014).

For the JM treatment of MiaPaCa-2 cell line subcutaneous xenografts, 100 µL of 1 million cells resuspended in 50% Matrigel/50% DMEM were implanted subcutaneously into the flank of 6–8-week-old nude mice. Once tumors were palpable, mice were randomized into treatment groups (control (4 mice) and JM (4 mice)) and treated intraperitoneally, with 1 mg/kg of JM twice daily for five times per week. Tumor size was monitored using caliper measurement. Each mouse was euthanized, and its tumor was harvested when it became moribund.

For the JM treatment of MiaPaCa-2 pancreatic orthotopic xenografts, mice were anesthetized with isoflurane, and meloxicam oral analgesia was used. A small abdominal flank incision was made and, using the spleen as the anchor, the pancreas was exteriorized. Next, 40 µL of 2 million cells resuspended in 50% DMEM/50% Matrigel were injected into the tail of the pancreas using a 28-gauge syringe needle. The needle was held in place for 30 s, and the injection site was swabbed with sterile gauze to prevent the leaking of tumor suspension. The pancreas was placed back into the peritoneal cavity, and the incision was sutured. Animals were monitored daily for 1 week post-injection to assess their overall health and wound healing. Mice were then imaged by ultrasound once per week with a FujiFilm VisualSonics Vevo 2100 high-frequency ultrasound (Toronto, ON, Canada). For KPC7107, 40 µL of 5000 cells was injected into C57BL/6. In the orthotopic MiPaCa-2 and KPC 7107 studies, the animals were divided into two treatment groups: control (4 mice) and JM (4 mice).

### 2.8. Western Blots

Cells were lysed in AB Lysis buffer, and Western blot analysis was performed. Blots were visualized and bands quantified using an Odyssey imaging system (LI-COR Biosciences, Lincoln, NE, USA). Primary antibodies’ total MYC (Y69 1:1000, Abcam, Waltham, MA, USA; ab32072), pMYC (1:500, Abcam, Waltham, MA, USA; ab78318), and GAPDH (6C5; 1:5000) were diluted in blocking buffer (1:1 Odyssey Blocking Buffer (LI-COR Biosciences, Lincoln, NE, USA)) PBS with 0.05% Tween20. Primary antibodies were detected with secondary antibodies labeled with the near-infrared fluorescent dyes IRDye800 (Rockland, Philadelphia, PA, USA) and Alexa Fluor 680 (Molecular Probes, Eugene, OR, USA) diluted 1:10,000 in a blocking buffer.

### 2.9. Quantitative RT-PCR (qRT-PCR)

RNA was isolated using an RNeasy kit with on-column DNase treatment (Qiagen, Germantown, MD, USA). cDNA was generated using the Multiscribe Reverse Transcriptase kit (Thermo Fisher, Waltham, MA, USA). qPCR analysis was performed with Fast SYBR Green reagent (Invitrogen, Waltham, MA, USA) using a StepOne machine (Applied Biosystems, Waltham, MA, USA). Primers were validated by performing a standard melt curve analysis, and the results are listed in the Key Resources Table.

### 2.10. Immunohistochemistry, Immunofluorescence, and Imaging

H&E staining was performed using standard methods with hematoxylin (Vector Laboratories, Burlingame, CA, USA) and Eosin Y solution (MilliporeSigma, Burlington, MA, USA). According to the manufacturer’s protocol, Masson’s Trichrome staining was performed with a Trichrome Stain Kit (MilliporeSigma).

For immunohistochemistry, formalin-fixed, paraffin-embedded (FFPE) sections were de-paraffinized and rehydrated, and antigen retrieval was performed in either pH 6 citric or pH 9 Tris-EDTA buffer (Dako) in a pressure cooker for 10 min. After cooling for 20 min, slides were quenched in 3% hydrogen peroxide for 10 min and blocked with 5% goat serum and 2.5% BSA for 1 h at room temperature. Primary antibodies Ki67 (G8; 1:400), TUNEL(MilliporeSigma™ Chemicon™ ApopTag™ Plus Peroxidase In Situ Apoptosis Kit: S7101), and pMYC (4B12 1:300) [30] were diluted in blocking buffer and tissues incubated overnight at 4 °C. Sections were incubated with anti-biotin secondary antibodies (1:1000) for 1 h, the Vectastain ABC kit (Vector Laboratories) for 1 h, and then exposed to the DAB substrate for 5–10 min for color development. Slides were counterstained with hematoxylin for 5 min and then mounted using Vectamount mounting media (Vector Laboratories).

For IF on FFPE tissue sections, slides were de-paraffinized and rehydrated, and antigen retrieval was performed using either a pH 6 citric or pH 9 Tris-EDTA-based buffer (Dako) in a pressure cooker for 10 min. After cooling for 20 min, slides were blocked with 10% goat serum and 1% BSA. Conjugated Primary antibodies Ki67 (CST:D3B5, 1:400), cleaved caspase-3 (CST:9661S, 1:50), Vimentin (CST:9854, 1:100), aSMA (SC:32251, 1:200), CD45 (CST:D3F8Q, 1:50), F480 (Biolegend:916104, 1:50), CollV (BDBiosciences:203003;1:100), PDPN (Biolegend:916606,1:100), CD8 (eBio:4SM15,1:100), CD11b (abcam:EPR1344,1:100), CD11c (CST:D4E3M,1:100), and pMYC (Sears lab, 4B12; 1:300) were diluted in blocking buffer and tissues incubated overnight at 4 °C. Slides were mounted with a SlowFade mounting reagent using DAPI. H&E and Trichrome images were acquired using an Aperio AT Scanscope with ImageScope (Leica Biosystems) software or a Zeiss AxioScan with Zeiss Zen software (Zeiss Microscopy, Thornwood, NY, USA). Images were acquired using a Zeiss AxioScan with Zeiss Zen software for the immunostaining of tissue sections. Tiled images were digitally stitched on Zen software to generate full scan images and analyzed with FIJI (ImageJ).

The quantification of IHC staining for pMYC and total MYC in tumors was performed through the deconvolution of the IHC image using ImageJ Fiji software version 2.1.0/1.53c.Color Deconvolution with the “H DAB” vector option to separate the hematoxylin staining (blue/purple) from the DAB staining (brown). The threshold was adjusted for the DAB staining image, and the % positive area was calculated by dividing the DAB positive area by the total area of the image. For the MiaPaCa-2 xenografts, 4 tumors of each type were analyzed. For the KPC tumors, 3 tumors were analyzed. At least 8 randomly selected ROIs were analyzed in each tumor.

### 2.11. Flow Cytometry

On day 18 of treatment, KPC7107 tumors (n = 3 per treatment group) were harvested in 1× PBS, minced to fine fragments, and incubated with Collagenase IV (Millipore Sigma, St. Louis, MO, USA), DNase I, Hyaluronidase, and a trypsin inhibitor (Sigma Aldrich, St. Louis, MO, USA) for 45 min at 37 °C on a rotating shaker. Enzymatic digestion was ceased by adding DMEM media containing 10% FBS. The resulting tissue homogenates were filtered through 70 μm cell strainers, and single-cell suspensions were collected and counted. Cell suspensions were then incubated with fixable viability dye-live dead Aqua (Thermo Fischer, L34966, 1:500) to gate viable cells. Non-specific antibody binding was blocked following incubation with the rat anti-mouse CD16/CD32 mAb (BD Bioscience, 553142, 12.4G2, 10 μg/mL) for 10 min at room temperature. Approximately 1 × 10^6^ cells per sample were labeled with the various fluorochrome-conjugated antibodies, washed, and resuspended in 2% FBS, 1× PBS buffer. The anti-mouse antibodies used in the experiment are the following; CD4-BUV496 (BioLegend, San Diego, CA, USA, BDB612952, GK1.5), CD8-BUV395 (BioLegend, 135011, A7R34), B220-APC (BioLegend, 400511, RTK2758), CD8a-BUV395 (Biolegend, BDB563786, 53–6.7), Ly6C-PerCP (BD Bioscience, 12808, HK1.4), CD45-BV421 (Fischer Scientific, 50-604-825, 30-F11), Ly6G-APC (Fischer Scientific, 50-164-399, 1A8), CD3-APC-efluor 780 (BD Bioscience, 100347, 145-2C1), CD11b-Superbright 600 (eBioscience, San Diego, CA, USA, 63-0112-80, M1/70), CD11c-PE (BioLegend, 108407, RB6-8C5), F4/80-APC (BioLegend, 123116, BM8), CD45-FITC (BioLegend, 141720, C068C2), and MHCII-AF700 (eBioscience, eB14-5321, M5/114.15.2). Flow cytometry data were obtained using a Cytek Aurora flow cytometer and analyzed using FlowJo software. The data presented are representative of singlet live cells.

### 2.12. RNA-Sequencing

For RNA-sequencing, RNA was isolated from MiaPaCa-2 pancreatic orthotopic xenograft tumors using an RNeasy kit with on-column DNase treatment (Qiagen), and RNA integrity was assessed using an Agilent 2100 Bioanalyzer with an RNA 6000 Nano Chip. RNA integrity numbers (RINs) were calculated from Bioanalyzer electropherograms using the “Eukaryotic Total RNA Nano” program of the Bioanalyzer 2100 Expert software (B.02.08.SI648). RIN values were in the 8.5–10 range, indicating high-quality RNA. Library preparation and sequencing were performed using the OHSU Massively Parallel Sequencing Shared Resource (RRID SCR_009984). Briefly, cDNA libraries were prepared from poly(A)-selected RNA using an Illumina TruSeq Stranded mRNA library preparation kit (Illumina, San Diego, CA, USA). Poly(A)+ RNA was isolated from 100 ng of total RNA (per sample) using oligo-dT-coated magnetic beads, which were then chemically fragmented. This is followed by the generation of first-strand cDNA using random hexamers as primers for reverse transcriptase and the subsequent synthesis of strand-specific second-strand cDNA with the addition of a single ‘A’ nucleotide to each end. Illumina adaptors were then ligated to the cDNAs. A 15-cycle PCR was used to amplify the material to yield the final libraries. Library concentration was determined using real-time PCR with primers complementary to the Illumina adaptors. Sample libraries were diluted and applied to an Illumina HiSeq 2500 Sequencer with a target of 50 M reads per sample, which was then used to assemble the reads into standard FastQ formatted data. Trimmomatic (version 0.39) [31] was used to trim low-quality reads and adapters (ILLUMINACLIP:2:30:10 SLIDINGWINDOW:5:25 LEADING:20 TRAILING:20 MINLEN:50). Fastqc (version 0.11.9) was used to assess the quality of trimmed reads. Only reads with forward and reverse pairs surviving were used for mapping. Mouse (GRCM39, refseq GCF_000001635.2) and human (GRCH38, refseq GCF_000001405.40) genomes were downloaded from NCBI and concatenated. This merged genome was then indexed and used to map trimmed reads using STAR (version 2.7.6). Reads aligning to mouse genes were removed from the count matrix. DESeq2 (version 1.34.0) [32] was used to examine the differential expression of human-mapping genes. Visualizations were produced with ggplot2 (version 3.4.2) and EnhancedVolcano (version 1.12.0).

We used gene set enrichment analysis (GSEA) [33] with default settings (1000 permutations) on the gene list obtained from DESeq data [32] to compare enrichment to gene ontology and hallmark gene sets in the MSigDB database (version 7.5.1) [34] to identify the pathways enriched by JM treatment. GSEA analysis was performed using the standalone GSEA application (version 4.3.2) using default parameters with normalized counts produced by using DESeq2 as the input. The hallmark gene set from MSigDB (version 2023.1) was used for pathway annotation. The statistically significant pathways were defined with a cutoff FDR < 0.25. In the setting of exploratory discovery, an FDR of 25% indicates that the result is likely to be valid 3 times out of 4, which is reasonable when one is interested in finding a candidate hypothesis to be further validated as a result of future research.

To find pathways and genes correlated with EGR1 expression in human PDAC data, we analyzed a pre-existing RNA-seq dataset [35] that includes 218 primary human pancreatic tumor profiles. Sample collection, processing, and sequencing is described in the dataset [35], and samples were obtained from the Oregon Pancreas Tissue Registry under IRB study # IRB00003609. Transcript abundances for the 218 primary tumor RNA-seq profiles were first summed into gene-level expression data using the R package tximport (v1.22.0) [36], then organized into a DESeq2 object to which we applied variance-stabilizing transformation (VST). To perform GSVA [37], we imported MSigDB hallmark gene sets via msigdbr (v.7.5.1) [34] and applied GSVA on VST count data with the GSVA R package (v.1.42.0).

### 2.13. Data and Statistics

Three or more independent biological replicate experiments were performed in all cases except those indicated in the figure legend. All standard errors were calculated from biological replicates. For all work except sequencing analyses, GraphPad Prism software was used for statistical analysis; individual tests and *p* values are described in the figure legends. For all experiments, the sample size was estimated based on a two-sample t-test with 1% significance level. The selected sample size was expected to provide 80% power to detect a mean difference of a 1.0 standard deviation unit.

### 2.14. Data Accessibility

RNA-seq reads have been deposited in NCBI BioProject under the accession number PRJNA1014468.

## 3. Results

### 3.1. Formulation and Characterization of JM

**Figure 1:**
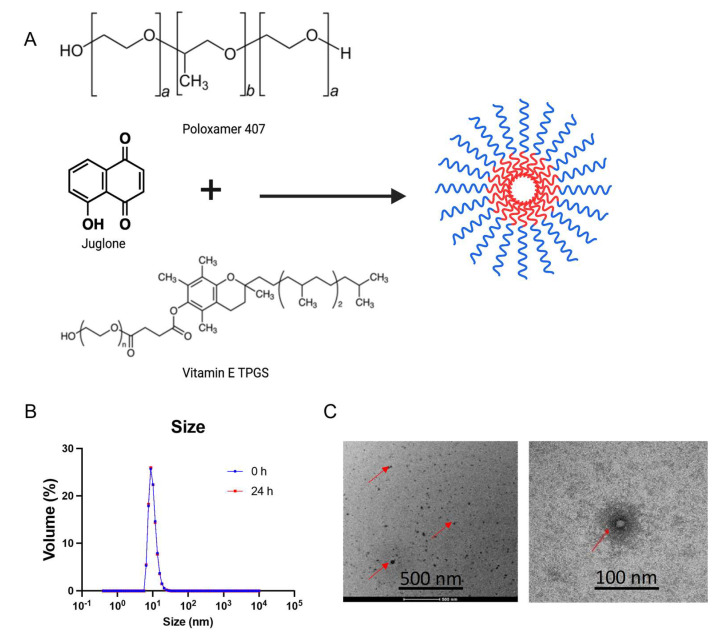
Synthesis and physicochemical characterization of juglone-loaded micelles (JMs): (**A**) Scheme depicting the procedure for the synthesis of Poloxamer 407 and vitamin E TPGS micelles as a carrier for delivery of juglone. The micelles were synthesized using the film hydration method. Poloxamer 407 and vitamin E TPGS at a ratio of 1:3 with juglone were added to ethanol and evaporated under a vacuum to form a drug-mixed polymeric micelle film. The film is then hydrated with deionized water/saline and filtered through a 0.22 µm polycarbonate filter to separate the nonencapsulated drug. Created with BioRender.com. (**B**) Size distribution of JMs determined using dynamic light scattering (DLS) at 0 h and 24 h. (**C**) Transmission electron microscopy (TEM) images of JMs. The images demonstrate a particle size of ~20 nm, equivalent to the DLS measurement. Data are shown as the mean ± SD (n = 3 observations).

JM was prepared by mixing F-127 and TPGS in 100% ethanol (Figure 1A). The ethanol in the mixture was evaporated using a rotatory evaporator, and the polymer and juglone were resuspended using MilliQ water passed through a 0.22 µm filter to form JM. The micelles had an average size of 16.2 ± 0.33 nm, a polydispersity index (PDI) of 0.14 ± 0.03, and a neutral zeta potential of −6.18 ± 0.29 mV at pH 7.4 (Figure 1B). Particles with a hydrodynamic diameter ≤100 nm have advantages in facilitating the efficient dispersion and absorption of drugs within tumors with high permeability [38]. However, scientific studies have demonstrated that only polymeric micelles with a particle size below 30 nm can infiltrate hypovascular and desmoplastic PDAC tissues, leading to a favorable antitumor effect [38]. The DLS measurements also showed unimodal size distribution as PDI < 0.2. Further, to assess the micelle formulation’s morphology and particle size, transmission electron microscopy was performed after staining with 1% uranyl acetate (Figure 1C). The drug loading efficiency was measured to be 50 ± 2.5% using high-performance liquid chromatography at a UV absorbance of 250 nm (method development: Supplementary Information). Further stability characterization of the micelles indicated that the micelles were stable for 24 h at room temperature with no signs of precipitation in the solution, and the size and PDI resembled the original values.

### 3.2. JMs Suppress Cell Proliferation Migration and Increase Apoptosis In Vitro

**Figure 2:**
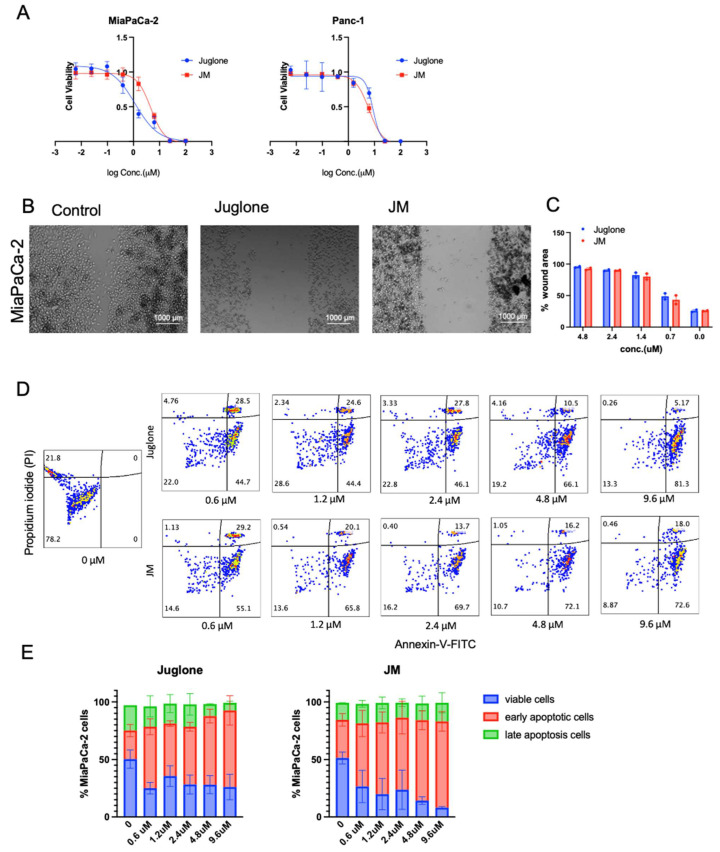
Juglone and JM suppress proliferation, migration, and enhance the apoptosis of pancreatic cancer cells: (**A**) MiaPaCa-2 and Panc-1 cells were seeded at 3000 cells/well density and exposed to various concentrations of juglone and JM for 48 h, followed by MTS assay. MTS assays indicated that both cells’ proliferation capacity was lower after treatment with various concentrations of juglone and JM. Data are presented as mean ± SD (n = 3). The data for the MTS assay are shown from 3 parallel experiments and are presented as mean ± SD. (**B**,**C**) Inhibition of the migration of MiaPaCa-2 cells as determined using a wound healing assay. Cells were treated with various drug concentrations of juglone and JM for 24 h. The images shown are representative images of cells treated with PBS, juglone (3 μM), and JM (3 μM) at 24 h. Data are presented as mean ± SD (n = 2). Statistical significance is determined using one-way ANOVA with Bonferroni’s multiple comparison test. (**D**,**E**) Evaluation and quantification of in vitro antitumor efficacy of juglone in Mia-PaCa-2 cells through Annexin V-FITC/PI staining. MiaPaCa-2 cells were treated with juglone and JM at the indicated concentrations for 24 h, followed by detecting and quantifying apoptosis using an FITC-Annexin V assay. The data are presented as the mean ± SD (n = 2).

To assess whether human pancreatic cell lines are sensitive to juglone and whether JM exhibited similar effects as the unformulated compound, we treated MiaPaCa-2 and Panc-1 human pancreatic cancer cell lines with various concentrations of both juglone and JM for 48 h followed by an MTS assay (Figure 2A). We found that juglone and JM decreased cell viability in both cell lines dose-dependently. The IC50 values were 6.6 ± 1.2 µM and 8.1 ± 1.4 µM for juglone in MiaPaCa-2 and Panc-1, respectively, whereas JM exhibited an IC50 of 8.1 ± 0.7 µM and 13.4 ± 1.7 µM in MiaPaCa-2 and Panc-1, respectively (Figure 2A). The MiaPaCa-2 cell line was slightly more sensitive to juglone and JM. Furthermore, the effect of juglone and JM on cell migration was investigated, as migration is critical in the progression of cancer and the spread of cancer cells to distant areas. A scratch was formed in the confluent monolayer of cells, and the cells were subsequently treated for 24 h with unformulated juglone and JM. The results of our investigation indicate that the migration of MiaPaCa-2 cells was significantly reduced by approximately 2–4-fold when exposed to treatment with either free or formulated juglone at concentrations below their IC50 values compared to untreated cells. The representative images depicting this observation are presented in Figure 2B. The results of the study showed that both juglone and JM reduced cell migration at 0.6, 1.2, 2.4, and 4.8 µM concentrations (Figure 2C). We saw no statistically significant difference in the cell migration with juglone and JM, indicating the formulation did not alter the biological function of juglone. Further investigation was conducted to examine the impact of juglone and JM on apoptosis and cell death in MiaPaCa-2 cells. Flow cytometric analysis was employed, utilizing Annexin V-FITC/Propidium iodide (PI) staining, as depicted in Figure 2D. The basis of the Annexin V apoptosis assay lies in the arrangement of phospholipids on the cell membrane, particularly phosphatidylserine. Apoptosis induces the externalization of phosphatidylserine, which has a high affinity for Annexin V, a protein with anticoagulant properties, which is then detected via flow cytometry on apoptotic cells. Propidium iodide (PI) is impermeable to membranes and rarely penetrates viable cells. Thus, Annexin V (−)—PI (−) represents healthy cells, Annexin V (+)—PI (−) indicates early apoptosis, and Annexin V (+)—PI (+) indicates cell necrosis and late apoptosis. The administration of juglone and JM for 24 h resulted in a dose-dependent increase in apoptotic cells (Figure 2D,E).

### 3.3. Unformulated Juglone Displayed Dose-Limiting Toxicity In Vivo

Juglone was dissolved in a minimal amount of ethanol to determine the maximum tolerated dose (MTD) for in vivo experiments, and the desired concentration was achieved by supplementing saline with 20% PEG. Juglone was administered via intraperitoneal injection to a sample size of two mice at varying dosages of 10 mg/kg, 5 mg/kg, 2.5 mg/kg, 1.25 mg/kg, and 1 mg/kg. The mice exhibited mortality within a time frame of 24 h at all concentrations except for at concentrations of 2.5, 1.25, and 1 mg/kg. Upon examination of the rodents, it was noted that a discernible cutaneous response was present at the injection site. Additionally, during the dissection process, we observed a similar reaction in the peritoneal cavity of rodents that were administered doses of 10 mg/kg and 5 mg/kg. The mice were subjected to a dosage of 2.5 mg/kg juglone, administered at a frequency of once every 24 h over 48 h. Following this, the mice demonstrated decreased body weight and presented with adverse effects on the skin. The mice were administered doses of 1.25 mg/kg and 1 mg/kg, respectively, at a frequency of once every 24 h for four days. The mice exhibited weight loss exceeding 10% of their body weight, displayed a hunched posture, and exhibited signs of illness, leading to the need for euthanasia six days after administering the initial dose. According to a previous study [39], animals that were administered a single dose of 3 mg/kg juglone exhibited a mortality rate of 20%, along with significant adverse effects. In that study, animals that received a single dose of 10 mg/kg juglone demonstrated a mortality rate of 100%, which aligns with the results obtained in our investigation. Previous research has presented evidence of administering doses as high as 5 mg/kg every 3 days for a duration of 30 days [40] or 1 mg/kg every other day for a period of 14 days [40]. However, our study encountered limitations in administering animals with a dosage exceeding 1 mg/kg daily over one week. As a result, it was concluded that the dosage of 1 mg/kg for juglone was ideal, aligning with previous research [39]. However, the administration of this dosage for a duration exceeding one week was not feasible due to the occurrence of systemic toxicity. Based on the aforementioned finding, we conducted an experiment involving formulated juglone. Our observations revealed that the formulated juglone could be effectively administered at a dosage of 1 mg/kg twice daily without any observable adverse effects on the skin or on weight loss. In light of these findings, all in vivo studies were exclusively conducted using formulated juglone. It is important to acknowledge that this approach represents a limitation of the study. However, the decision to exclude juglone as a separate arm was based on its inherent toxicity, which would have compromised the scientific objectivity of the investigation.

### 3.4. JM Inhibits Subcutaneous and Orthotopic Pancreatic Tumor Growth In Vivo

**Figure 3:**
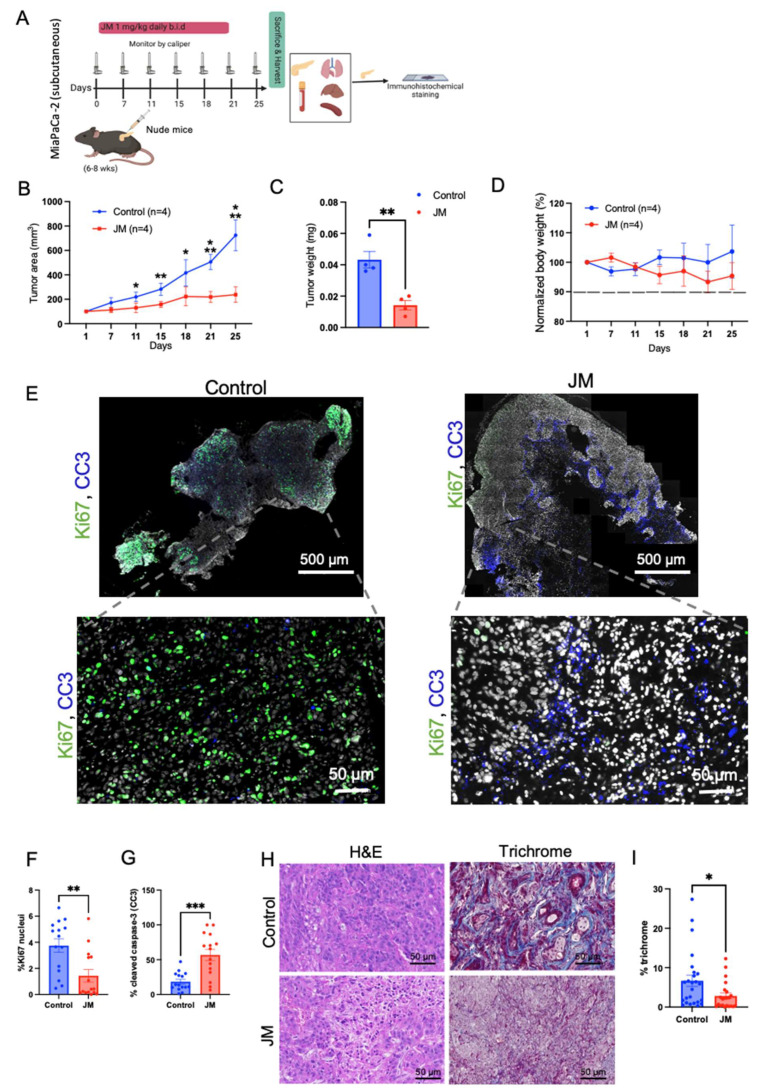
In-vivo antitumor efficacy of JM in immunocompromised mice: (**A**) study treatment protocol. Animals were injected with MiaPaca-2 pancreatic cancer cells subcutaneously; once the tumors had reached a volume of 50 mm^3^, the mice were randomly assigned to either the control group or the group receiving JM. The mice received daily intraperitoneal injections of 1 mg/kg of JM twice daily for 25 days of activity. (**B**) Tumors were calipered, and the tumor volume was plotted across time for control and JM groups (n = 4). Mean ± SEM shown. AUC was analyzed for each treatment arm, *p* < 0.05, using a one-way ANOVA. (**C**) Quantification of the endpoint tumor size from B. Mean ± SEM shown (n = 4). (**D**) Variations in body weight. Data are presented as mean ± SD, n = 4 mice. MiaPaCa-3 xenograft tumors were grown as in A and harvested after 25 days of treatment with saline, JM (1 mg/kg, b.i.d). (**E**) Tissue sections were assessed for cell proliferation using Ki67 staining and cell death using cleaved caspase-3 (CC3) staining; the representative images for control and JM-treated tumors are shown (n = 3). (**F**) Percent Ki67 and (**G**) percent CC3, quantified using ImageJ on control and JM-treated tumors (n = 3). (**H**) H&E and trichrome staining (collagen deposition). (**I**) Percent collagen quantified from the trichrome staining from xenograft pancreatic cancer tumors treated with saline or JM. Data are presented as mean ± SEM. Statistical analyses were performed using the unpaired parametric Welch t-test to compare the means between two independent groups. * *p* < 0.05, ** *p* < 0.01, and *** *p* < 0.001. (**A**) Created with BioRender.com.

A subcutaneous tumor model was initially employed to assess the in vivo antitumor efficacy of JM. This model was chosen due to its inherent advantages, including the ability to induce tumor growth without the need for surgical intervention and the convenience it offers for accurate tumor measurements. The MiaPaCa-2 cell line was introduced into the subcutaneous region of immunocompromised mice. Once the tumors had reached a volume of 50 mm^3^, the mice were randomly assigned to either the control group or the group receiving JM treatment. The administration of JM treatment involved a dosage of 1.0 mg/kg administered twice daily via an intraperitoneal injection over a period of 25 days; the treatment protocol is shown in Figure 3A. This dosing frequency was chosen to maximize the juglone’s therapeutic effect, considering the short half-life of micelles in circulation and the juglone’s limited circulation time. By administering JM twice daily, we aimed to increase the likelihood of juglone reaching tumor cells, thus enhancing its therapeutic efficacy.

In contrast, the control group of mice received a similar treatment regimen using saline solution. The administration of JM resulted in the suppression of tumor growth and a reduction in final tumor weight, as depicted in Figure 3B,C. Notably, no significant change in body weight was observed in either the control or experimental groups, as illustrated in Figure 3D. These findings indicate that the JMs did not exhibit any discernible systemic toxicity. The results presented in Figure 3E,G demonstrate that the JM-treated tumors resulted in a notable increase in apoptosis, as indicated by the presence of cleaved caspase 3 (CC3). Additionally, there was a decrease in proliferation, as evidenced by significantly reduced levels of Ki67, as depicted in the representative images in Figure 3E,F.

The presence of a distinct desmoplastic microenvironment is a characteristic feature of PDAC that has significant implications for the initiation, progression, and metastasis of tumors [41]. Collagen is the primary component of the extracellular matrix (ECM) that contributes to the desmoplastic characteristic of PDAC. Enhanced collagen buildup leads to increased interstitial fluid pressures, which induce blood vessel compression and reduce the efficacy of chemotherapeutic drug delivery. Through the trichrome staining of the tumor tissues, we observed low collagen density (~three-fold compared to the control group) throughout the tumor lesion in the JM-treated group (Figure 3H,I). The mechanism underlying the observed reduction in collagen levels in the group treated with JM remains inadequately understood and necessitates further investigation. However, it is reasonable to attribute this phenomenon to the comparatively smaller tumor sizes observed in the treatment group compared to the control group. Nevertheless, this observation aligns with prior studies conducted in this area, which have established that nanoformulations can alter the tumor microenvironment, potentially increasing cytotoxic effects [42,43]. In general, the data indicated that JM demonstrated significant antitumor efficacy while exhibiting lower levels of toxicity compared to juglone, as evidenced by the absence of weight loss and the overall health of the observed mice.

**Figure 4:**
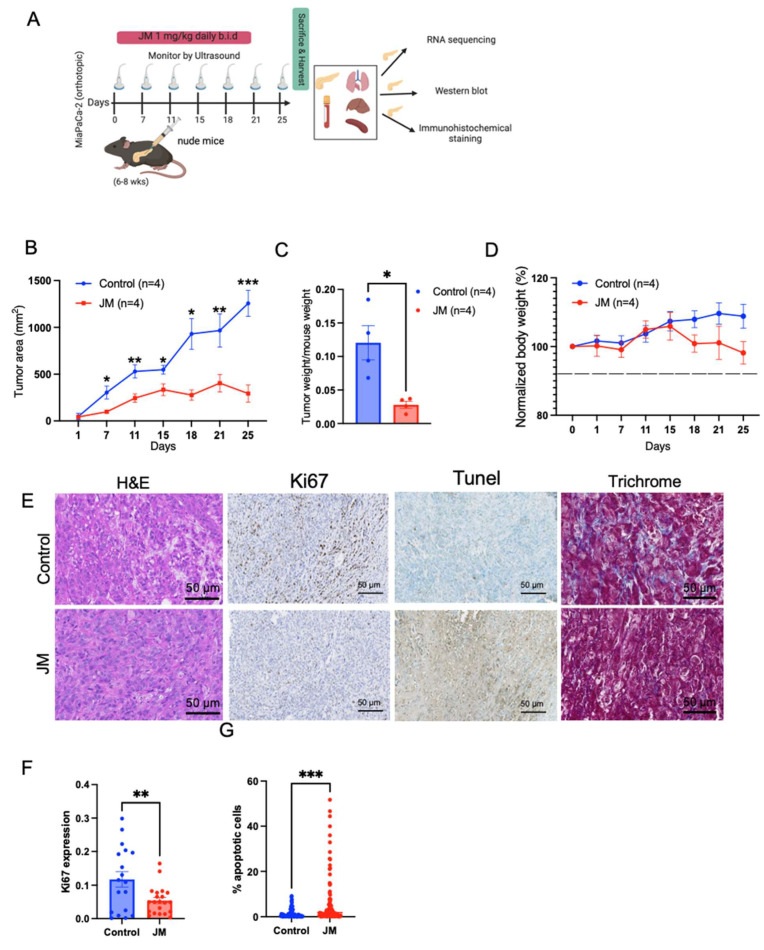
JM inhibited the growth of orthotopic MiaPaCa-2 human pancreatic cancer xenografts: (**A**) Illustration of the establishment of pancreatic tumors and treatment schedule. (**B**) Changes in tumor burden as assessed and confirmed through ultrasound imaging using the 3D B-Mode of the Vevo 2100 ultrasound system. Data are presented as mean ± SEM (n = 4). (**C**) Quantifying tumor weight at the experimental endpoint (day 25) normalized to body weight from B. Mean ± SEM shown (n = 4). (**D**) Body weight changes throughout the experiment. Tissues harvested after 25 days of treatment with juglone and JM were sectioned and stained for (**E**) histology using H&E staining, cell proliferation using Ki67 staining, cell death using TUNEL staining, and collagen using trichrome staining. (**F**) Percent Ki67 and (**G**) percent apoptotic cells (TUNEL) were quantified using ImageJ on control. JM-treated tumors (n = 3). * *p* < 0.05, ** *p* < 0.01, and *** *p* < 0.001.

To evaluate the therapeutic efficacy of JM in addressing the tissue microenvironment (TME) of pancreatic tumors, the in-vivo effectiveness of JM was examined using an orthotopic MiaPaCa-2 tumor model. The treatment protocol employed in this study is depicted in Figure 4A. The results presented in Figure 4B,C indicate that the administration of JM significantly reduced tumor burden compared to the control group of mice. This observation was validated through ultrasound measurements conducted throughout the study, and the final tumor weight was recorded at the endpoint (Figure 4B,C). Furthermore, it was observed that there was no decrease in body weight throughout the duration of the treatment, indicating that the JM was well tolerated (Figure 4D). In accordance with our in vitro and subcutaneous model, we observed an elevation in the number of Tunel-positive cells (indicating increased apoptosis) and a reduction in the number of Ki67-positive cells (indicating decreased proliferation) in the group of mice treated with JM compared to the control group. This observation was consistent across H&E staining, Tunel, and Ki67 immunohistochemical staining (Figure 4E–G).

### 3.5. JM Treatment In-Vivo Strongly Impacts Essential Pro-Oncogenic Pathways, Including MYC Pathway and Gene Signature

To assess the impact of JM on the transcriptome and characterize molecular pathways that may inform the mechanisms that drive its therapeutic response, we conducted paired-end RNA sequencing (RNA-seq) and differential expression analysis on MiaPaCa-2 orthotopic tumors grouped as both control and JM.

**Figure 5:**
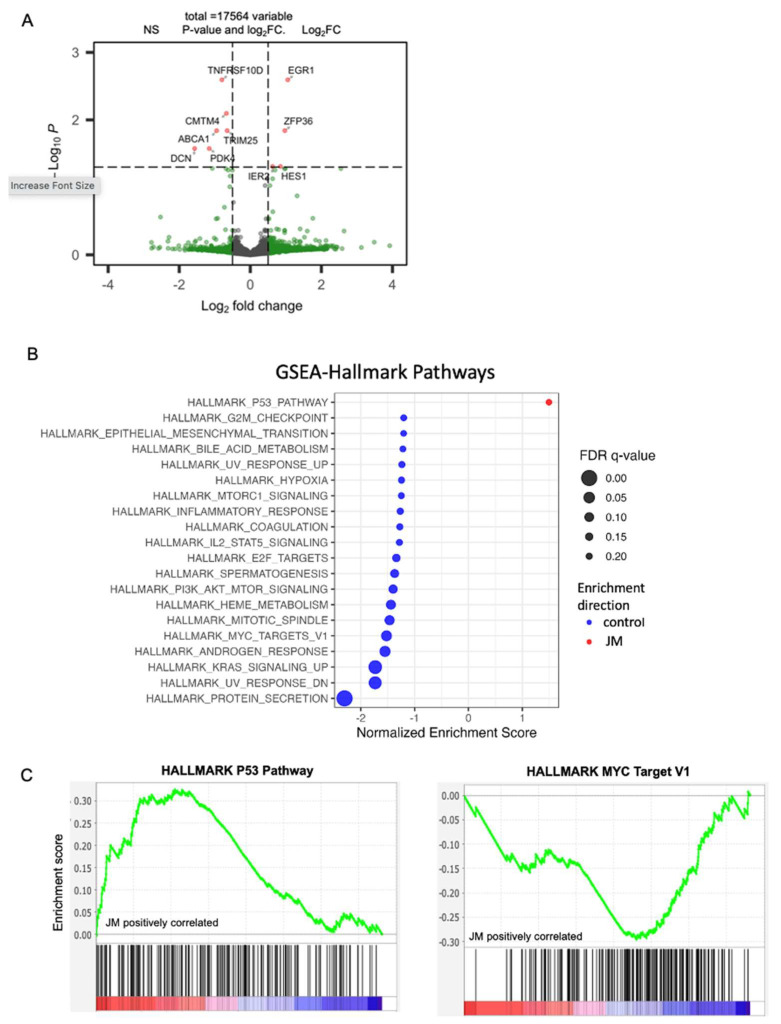

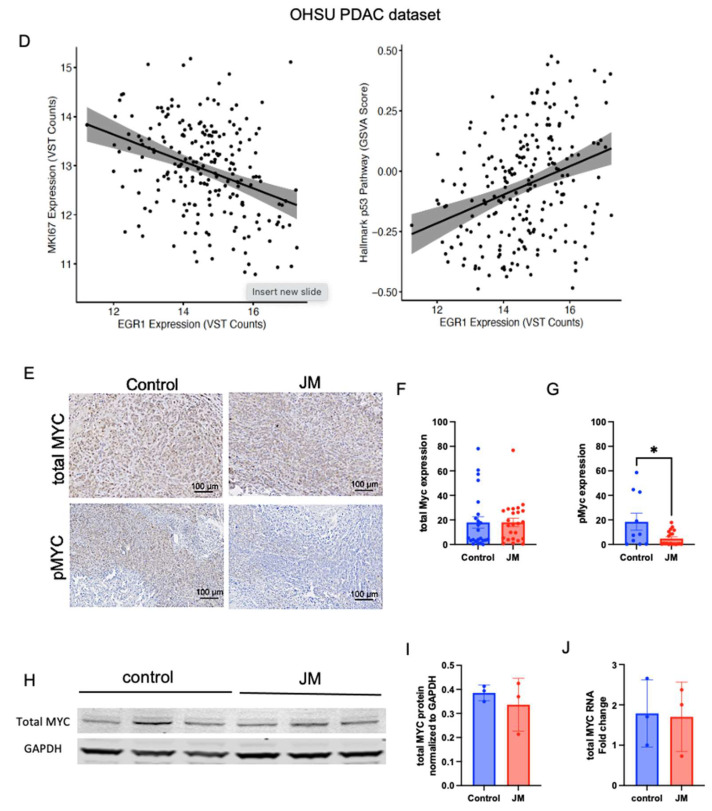
(**A**) Volcano plots of statistical significance (-log10 adjusted *p*-value) against fold change (log2) of genes expressed in orthotopic control vs. JM-treated tumors. Significantly upregulated genes with a *p*-value of 0.05 and log2FC are shown in red; only log2FC are shown in green, and no significant (NS) genes are shown in gray. (**B**) Gene set enrichment analysis (GSEA) of orthotopic MiaPaCa-2-injected pancreatic cancer mice grouped as control and JM-treated tumors (1.0 mg/kg). Summary of selected pathways with FDR < 0.25 enriched in control vs. JM tumors. (**C**) The P53 hallmark pathway is downregulated in the control compared to tumors treated with JM, and the MYC_Targets V1 pathway is upregulated in the control compared to tumors treated with JM (n = 4). (**D**) GSVA analysis of EGR1 correlation to MKI67 (r^2^ = −0.35) and p53 (r^2^ = 0.32) pathways in the human PDAC dataset (n = 218 primary tumors). (**E**–**G**) Expression and quantification of total MYC and pMYC in orthotopic tumors with immunohistochemistry. (**H**,**I**). Assessment of total MYC at protein and RNA levels in control and JM orthotopic tumors (n = 3) (**I**,**J**). * *p* < 0.05.

The differential expression analysis showed that the expression of nine genes was much higher in the JM-treated tumors than in the MiaPaCa-2 control group. These nine genes had log2 fold changes greater than 0.5 or less than −0.5 and a false discovery rate (FDR)-adjusted *p*-value less than 0.05 (Figure 5A). These genes may be linked to the response to JM treatment and may shed light on putative molecular pathways implicated in the therapeutic response. Using gene-set enrichment analysis (GSEA) with cohorts from MSigDB (Figure 5B), we aimed to identify hallmark pathways that exhibited enrichment in the JM-treated group compared to the control tumors. GSEA revealed that the hallmark P53 pathway was upregulated in JM-treated tumors while multiple other hallmark pathways were upregulated in the control tumors based on FDR-adjusted p-values less than 0.2 (Figure 5B). Some of the hallmark pathways that were significantly upregulated in the control group included MYC signaling targets, the G2/M checkpoint, E2F targets, and epithelial–mesenchymal transition pathways (Figure 5B,C). These pathways play crucial roles in various biological processes, including cell proliferation, cell cycle regulation, and signal transduction. The enrichment of these hallmark pathways in the control tumors suggests that these biological processes may be inhibited in response to JM treatment (Figure 5C), potentially contributing to suppression of tumor growth. While GSEA suggests that molecular pathways in JM therapy may regulate and contribute to its therapeutic response, it emphasizes the need to study these pathways and genes to understand JM’s effects on tumor biology and its therapeutic potential.

Further, gene set variation analysis (GSVA) [37] was used to compare the most upregulated gene, EGR1, and other pathways using a human PDAC dataset 218 primary tumor samples [35]. The analysis revealed an inverse correlation between the expression of EGR1 and MKI67, a widely recognized marker for proliferation (commonly known as Ki67) (Figure 5D). This suggests that the increase in EGR1 after treatment with JM may be linked to a decrease in cell growth, which is reflected in the decline in MKI67. The GSVA analysis also found a link between the expression of EGR1 and the p53 pathway, which regulates apoptosis (Figure 5D). This finding suggests that EGR1 overexpression is linked to p53 pathway activation, which induces apoptosis and reduces tumor growth. These results are in accordance with the analysis of mouse tumors that were treated with JM. By integrating the results of the differential expression analysis, gene set enrichment analysis, and gene set variation analysis, this study provides an initial understanding of the molecular changes underlying JM therapy’s therapeutic effects on PDAC.

Additionally, the modulation of MYC expression in response to JM treatment was investigated. The MYC_targets_V1 oncogenic signature, which consists of genes under the control of the MYC oncoprotein, had higher expression levels in the control tumors than in the JM-treated tumors. This observation suggests that MYC-regulated pathways may be more active in control tumors and potentially associated with drug response. Immunohistochemistry was used to measure the levels of total MYC and phosphorylated MYC at Serine 62 (pMYC), a mark of transcriptionally active MYC [44,45], in orthotopic tumors to examine the modulation of MYC expression. While total MYC levels remain unaffected, tumors treated with JM displayed a significant decrease in pMYC levels relative to control tumors (Figure 5E–G). To validate this, we evaluated the levels of total MYC via Western blot analysis for protein (data normalized to GAPDH) (Figure 5I) and qRT-PCR (data normalized to 18s) for RNA (Figure 5J) in the tumors. The overall levels of total MYC with staining and at both protein and RNA levels showed no significant disparity between the control and JM-treated tumors (Figure 5I,J). These findings indicate that, while there was no significant difference in the overall levels of the total MYC protein between the control and JM-treated tumors, there was a notable decrease in the levels of pMYC in the JM-treated tumors. This suggests that JM treatment might impact MYC’s activation or phosphorylation status, potentially affecting its downstream signaling and functions. The fact that JM treatment led to a drop in pMYC levels is consistent with the results of differential expression analysis, gene set enrichment analysis, and gene set variation analysis, suggesting that JM treatment can affect the MYC pathway and its downstream targets. More research into how juglone controls MYC could help explain how therapy works and how pancreatic cancer cells might become resistant to drugs.

### 3.6. JM Attenuates PDAC Growth by Suppressing Immune Accumulation and ECM Modulation

**Figure 6:**
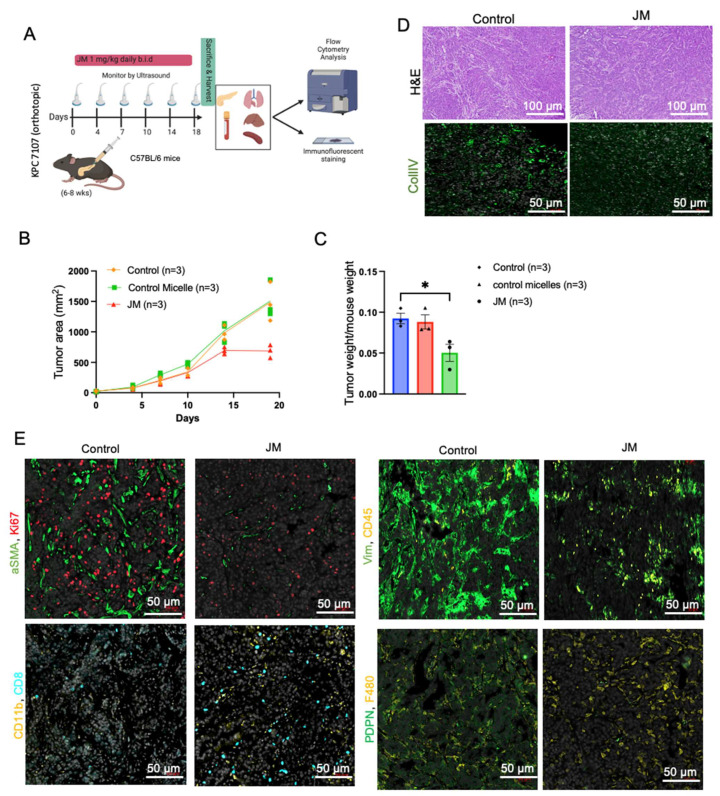

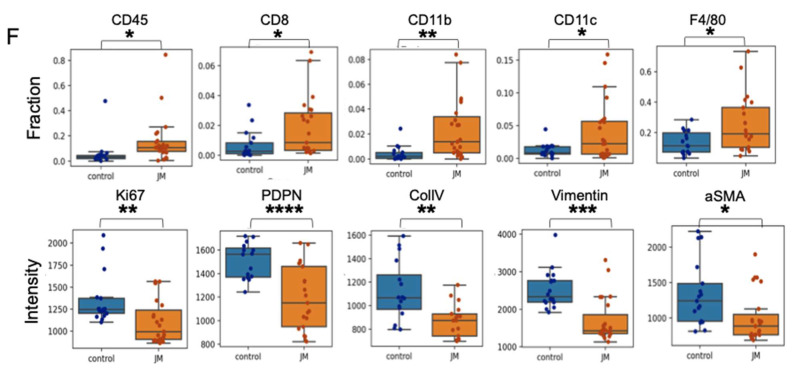
JM displayed increased antitumor activity compared to control micelles in the KPC mouse model: (**A**) The timeline of experiments on the KPC model. (**B**) Growth curve of an orthotopic pancreatic allograft of KPC7107 cell lines in C57BL/6 mice. The area was measured using an ultrasound using the 3D B-Mode of the Vevo 2100 system throughout 19 days; data are shown as (mean ± SEM, n = 3). (**C**) Tumor weight of PDAC in KPC mice treated with saline, control micelles, and JM (1.0 mg/kg, bid) (mean ± SEM, n = 3). (**D**) Histopathological analysis of the KPC mice tumors and representative collagen (CollIV) images using immunofluorescence staining. Paraffin-embedded KPC tumor tissues were stained with immunofluorescence. Representative images from both control and JM-treated tumors are shown in two panels. (**E**) Fibroblast activation marker (aSMA) and proliferation (Ki67), ECM marker (vimentin) and total immune cells (CD45) (upper panels), the presence of myeloid cells (CD11b) and T cells (CD8) and fibroblast marker (PDPN), and pan macrophage marker (F480) (lower panels) with DAPI staining nuclei (Scale bar: 50 μm). (**F**) Quantification of the immunofluorescent markers as shown in (**E**). Data are presented as the mean ± SD. * *p*  <  0.05, ** *p*  <  0.01 and *** *p*  <  0.001, **** *p*  <  0.0001.

Next, given JM’s effectiveness in reducing tumor growth in immunocompromised animals, we investigated its effect in immunocompetent mice. Figure 6A shows our treatment plan. In short, we injected LSL-KrasG12D/+, LSL-Trp53R172H/+, and Pdx-1-Cre (KPC) cells into the pancreas of healthy syngeneic mice. Twelve days later, we started treating the tumors that formed with a dose of juglone micelles twice per day for 18 days. After 30 days (Figure 6), mice were euthanized, and their tumors were processed for downstream analysis. PDAC tumors were substantially reduced in JM-treated mice compared to saline- or empty-micelle-treated controls. Mice that were given empty micelles had tumors that were, statistically, as big as those of the control mice. In this way, it was determined that empty micelles did not exert any therapeutic benefits.

PDAC tumors are generally characterized by an excessive deposition of ECM, deposited by activated fibroblasts, and a low degree of immune infiltration. Increased deposition of collagen, which is produced and sustained by cancer-associated fibroblasts (CAFs), results in increased interstitial fluid pressures that can cause the constriction of blood vessels and, therefore, act as a barrier to the efficient delivery of chemotherapeutics [46]. We stained the tumors for collagen (Figure 6C,E), the most prominent component of PDAC ECM, to determine if JM had any effect on these features of the PDAC tumor microenvironment. Collagen deposition was much lower in JM-treated tumors compared to control tumors (2-fold), which is consistent with the experiments shown in Figure 3H,I and Figure 4E. Additionally, immunofluorescence confirmed a comparable decrease in CAF activation markers, alpha-smooth muscle actin (α-SMA), vimentin (Figure 6D,E), and the overall number of CAFs stained with podoplanin (PDPN) (Figure 6D,E). The findings mentioned above indicate that the cytotoxic effects of JM may lead to a decrease in the number of PDPN-positive fibroblasts that express α-SMA (Figure 6D,E). In an alternative scenario, it is plausible that cancer-associated fibroblasts experience a transformation from an activated state to a quiescent state due to the JM treatments. Either of these mechanisms, collectively or individually, would contribute to reducing collagen accumulation within primary tumors, mitigate tumor fibrosis, and restore more normal ECM. Additionally, it is crucial to consider the potential influence of delayed tumor growth on the reduced recruitment of fibroblasts to the tumor tissue in animals treated with JM.

Considering PDAC as a low-immunogenic tumor with an immune-suppressed TME, immunofluorescent staining was performed on both control and JM-treated tumors to detect immune markers. Compared to the control group, the experimental group that received treatment with JM demonstrated elevated levels of diverse immune cell populations, including CD45+ immune cells, CD8+ effector T cells, CD11b+ myeloid cells, CD11+ dendritic cells, and F4/80+ macrophages (Figure 6D,E). This observation implies that the JM treatment may reverse the immunologically inactive state of the control tumors, known as the “cold” phenotype, and make them responsive to the immune system. Flow cytometry was used to corroborate these findings (Supplementary Figure S1). Although a similar trend was observed in the staining results, the statistical significance could not be determined for the entire dataset. The lack of a difference in significance may be attributable to the small number of cells and limited sample size used in the analysis.

In the context of PDAC, empirical evidence indicates a correlation between the deposition of ECM and diminished immune infiltration and the hindered delivery of therapeutic agents to tumor cells. Nevertheless, it has been noted that the deposition of ECM can potentially augment a more aggressive and invasive phenotype while simultaneously creating a microenvironment conducive to the proliferation of cancer cells [47].

## 4. Discussion

PDAC is characterized by a significantly low 5-year survival rate that has just recently reached 12%, indicating a poor prognosis for patients affected by this condition. This alarming statistic highlights the necessity for an innovative therapeutic approach. A growing body of research has compiled findings on the potential application of natural products as agents for inhibiting tumor growth. Natural products refer to the components derived from animals, plants, marine organisms, or microorganisms [48]. Furthermore, various chemotherapeutic drugs and their analogs, including vincristine and paclitaxel, have been artificially produced using natural substances. Juglone, a naturally occurring compound, has demonstrated cytotoxic effects against various cancer types. Mechanistically, juglone inhibits cancer cell proliferation by inhibiting stem cell properties, epithelial-to-mesenchymal transition, and angiogenesis [49], inhibiting cell cycle progression [14], and/or by inducing apoptosis via mitochondrial-dependent pathways [17]. Moreover, in vivo, juglone inhibited the progression of tumors in rodents by increasing apoptosis and cell cycle blockage [50,51].

While previous research, primarily conducted through in vitro experiments, has indicated that juglone has an impact on the proliferation, apoptosis, and metastasis of multiple cancer cell lines, there remains a need for further investigation into the role of juglone in vivo and in pancreatic cancer. In pancreatic cancer, juglone has been shown to exhibit inhibitory effects on the proliferation of PDAC cell lines through the induction of cell cycle arrest and apoptosis [11]. Clinical use of juglone is hampered by its physicochemical properties, particularly its low water solubility, despite its potential therapeutic usefulness as an anticancer therapy. Prior research has examined the effects of juglone on animal tumor models such as liver metastasis [13], melanoma [39,52], murine mammary adenocarcinoma [50], and colorectal cancer [53] utilizing free drug and liposomal formulations [54]. However, these studies were affected by toxicity that limited them to a small number of administered doses (a total of three or four doses) every other day. Furthermore, no studies have been performed to evaluate the in vivo effect of juglone in pancreatic mouse models.

To overcome the inherent toxicity issue, we formulated a mixed micelle formulation of juglone. The data showed that the micelle formulation did not alter juglone’s cytotoxic properties but increased its water solubility. Upon evaluating its effect on proliferation, migration, and apoptosis in pancreatic cancer cells, the micelles inhibited proliferation, migration, and increased apoptosis in pancreatic cell lines, which is consistent with the published effect of juglone in pancreatic cell lines and other cancer cell lines. Herein, we performed in vivo experiments on both flank xenografts in the orthotopic PDAC models in immunocompetent and immunocompromised mice. We show tumor burden reduction compared to the control animals, which is consistent with the abovementioned studies. Hence, juglone micelles resolved the in vivo application of juglone and enhanced antitumor efficacy. This effect was correlated with increased apoptosis and decreased proliferation in all models. We also increased the antitumor effect and the tolerability of juglone as no observed side effects, such as death or weight loss, existed in mice with JM.

PDAC exhibits remarkable resistance to conventional therapy compared to other cancers and possesses a highly immunosuppressive tumor microenvironment. To interrogate if the antitumor effect of juglone is mediated by immune function, we used the syngeneic mouse model with a KPC cell line. Our data shows that juglone micelles induced an increased level of CD45+ compared to the control group. Moreover, we observed an increase in CD8+ cells in the treatment group compared to the control group. Meanwhile, we also showed an increase in CD11b+ cells, suggesting an effect on myeloid cells. We also observed decreased fibroblast activation markers such as vimentin, αSMA, and ColIV, suggesting JM modulated the stromal compartment of pancreatic cancer. Our data indicate that juglone exerts antitumor activity by directly acting on the cancer cells and modulating the immune and stromal microenvironments of PDAC.

TME is a complex and dynamic ecosystem of cancer cells, stromal cells, immune cells, and extracellular matrix components. The interactions between these various cellular and molecular elements play a crucial role in tumor growth, progression, and response to therapy. Beyond its direct antitumor effects, the treatment of JM has demonstrated a unique capacity to influence TME in immunocompetent mice, adding a dimension to its therapeutic potential. The immune system plays a pivotal role in recognizing and eliminating cancer cells. However, tumors often employ various mechanisms to evade immune surveillance. We found JM to promote the infiltration of immune cells, such as T cells, into the tumor microenvironment. This phenomenon indicates that JM can modulate the immunosuppressive TME, creating a more favorable environment for the immune system to mount an antitumor response. The increased immune cell infiltration in response to JM treatment indicates an immunomodulatory role. By promoting the presence and activity of immune effector cells within the tumor, JM can enhance the recognition and elimination of cancer cells, leading to an improved antitumor immune response. The stromal components of the TME, such as CAFs and ECM proteins, contribute to tumor growth and progression. These elements create a physical barrier that hinders drug penetration and immune cell infiltration. JM treatment was associated with a reduced ECM deposition, suggesting that it may remodel the tumor stroma and improve drug and immune cell accessibility to cancer cells. Activation markers on immune and stromal cells are indicative of their functional state.

JM treatment downregulates specific pro-tumor activation markers in the TME, potentially indicating a reduction in these cells’ supportive or immunosuppressive activity. This downregulation could contribute to the establishment of a more immune-permissive microenvironment. The immunomodulatory effects of JM could potentially complement immunotherapeutic approaches, such as immune checkpoint inhibitors or adoptive T-cell therapies. Combining JM with immunotherapies may enhance their efficacy by sensitizing the TME and augmenting the antitumor immune response. Overall, the observed influence of JM on the tumor microenvironment provides a novel perspective on its therapeutic potential. By reshaping the immune landscape and stromal composition within the tumor, JM can create a more permissive environment for antitumor immune responses and improve the overall effectiveness of cancer therapy. These findings underscore the importance of considering the immunomodulatory properties of JM and its potential for combination therapies with immunotherapeutic agents in the quest for more effective and personalized cancer treatments.

Numerous studies have furnished findings in favor of the notion that specific chemotherapeutic agents, as well as their nanoformulations, possess the capacity to induce cytotoxic effects and play a role in the restructuring of the tumor microenvironment. For instance, the therapeutic potential of a nanoparticle formulation containing oxaliplatin is believed to be enhanced by its increased cytotoxicity, the elevated response of dendritic cells, and the more significant infiltration of CD8+ T cells. Additionally, using this nanoparticle formulation reduces toxicity compared to administering free oxaliplatin [55].

Moreover, incorporating a nitric oxide donor and recombinant tumor necrosis factor-related apoptosis-inducing ligand (TRAIL) into the nanogel formulation can modify the desmoplastic stroma and reduce activity in the antiapoptotic pathway. The proposed alteration can increase the tumor-penetrating ability of TRAIL and significantly improve the effectiveness of TRAIL therapy in tumor treatment [56]. While the exact mechanism behind the observed remodeling of the tumor microenvironment in response to JM requires further investigation, it is plausible that this phenomenon may contribute to the observed antitumor activity of this formulation. In summary, the data indicate that JM exhibits efficacy in inhibiting the advancement of PDAC by modulating the expression of markers linked to fibroblast activation and immune cells.

PDAC is a complex disease with various molecular alterations. Combining juglone with agents that target different signaling pathways can address the heterogeneity of the tumor and increase the likelihood of hitting multiple vulnerable points within cancer cells. Resistance to single-agent therapies is a common challenge in cancer treatment. With combination therapies, we can target multiple pathways simultaneously, making it more difficult for cancer cells to develop resistance. Combinatorial strategies can involve combining juglone with conventional chemotherapy, targeted therapies, immunotherapies, or other experimental agents. The choice of combination partners should be based on a solid understanding of the underlying molecular pathways driving pancreatic cancer growth, progression, and drug resistance. Based on our work, juglone’s immunomodulatory effects, when combined with immunotherapies or drugs that target the stromal components of the tumor, could enhance the overall antitumor response and promote a more favorable microenvironment for treatment. Identifying drugs that complement juglone’s mechanism of action could improve its ability to inhibit tumor growth, induce apoptosis, and suppress drug resistance. Combining juglone and other targeted agents or chemotherapy could help overcome resistance mechanisms and improve treatment outcomes. Moreover, based on our transcriptomics data, MYC might be a potential biomarker/target that can predict responses to juglone or other agents, enabling clinicians to design more effective and personalized treatment regimens.

## 5. Conclusions

In conclusion, our study highlights the significant anticancer effects of juglone, both in vitro and in vivo. It effectively inhibits cancer growth by suppressing proliferation, promoting apoptosis, activating the immune system, and normalizing the tumor microenvironment. These diverse properties make juglone an up-and-coming candidate for pancreatic cancer therapy. To enhance its antitumor effectiveness while reducing adverse effects, we utilized polymeric micelles as a drug delivery system for juglone. This approach proved successful in our preclinical models. While the exact mechanism of juglone-induced cytotoxicity in pancreatic cancer requires further investigation, our proof-of-concept study unequivocally demonstrates the potential of juglone for therapeutic management in PDAC. Combining juglone with other agents and novel drug delivery systems presents an exciting avenue for future research. JM shows excellent promise as a pancreatic cancer therapeutic, and combination therapies could further maximize its anticancer effects. Continued research and clinical trials are essential to advance personalized and effective treatment strategies for patients facing this challenging disease.

## Data Availability

Data is present in the article.

## Supplementary Materials

The following supporting information can be downloaded at: https://www.mdpi.com/article/10.3390/pharmaceutics15122651/s1, Figure S1: JM treatment modulates immune infiltration, which may help in the antitumor response.
