# Supplementary material for: Micelle-Formulated Juglone Effectively Targets Pancreatic Cancer and Remodels the Tumor Microenvironment
Source: Pharmaceutics. 2023 Nov 21;15(12):2651. doi: 10.3390/pharmaceutics15122651 (PMC10747591; doi:10.3390/pharmaceutics15122651)
Supplement: Supplementary file 1 [file pharmaceutics-15-02651-s001.zip › pharmaceutics-2637934-supplementary.pdf]

Supplementary information S1

Method development

Preparation of standard solution

Five milligrams of Juglone were accurately weighed and transferred to a glass scintillation vial. One milliliter of ethanol was added, and with sonication, it was dissolved completely. Two hundred microliters of aliquot were withdrawn and diluted to one milliliter with diluent (mobile phase) to obtain the working solutions.

The system consisted of reverse-phase high-performance liquid chromatography (RP-HPLC), (Shimadzu, USA). The analysis was performed using a Zorbax C18 column (4.6 × 75 mm, 3.5 μm) (Agilent Technologies, Santa Clara, CA) in an isocratic mode with acetonitrile/water (50/50) containing 0.1% phosphoric acid and 1% methanol, at a flow rate of 1.5 mL/min, and an injection volume of 10 μL. Column temperature was maintained at 40 °C with a run time of 3 min. The juglone peak is monitored at 250 nm and has a retention time of 1.5 min.
The developed method was validated for system specificity, linearity, precision, robustness, Limit of detection and Limit of quantification.

Specificity

This parameter assesses the ability of the method to measure the Juglone to be selective. It can accurately determine the Juglone in the presence of potential interfering substances. For example, the mobile phase should not interfere with the analysis of the Juglone for the developed method to be specific. The blank and juglone solution was injected, and no interference was observed at the juglone retention time of 1.5 min.

Chromatograms of (**a**) blank (**b**) juglone standard

a.

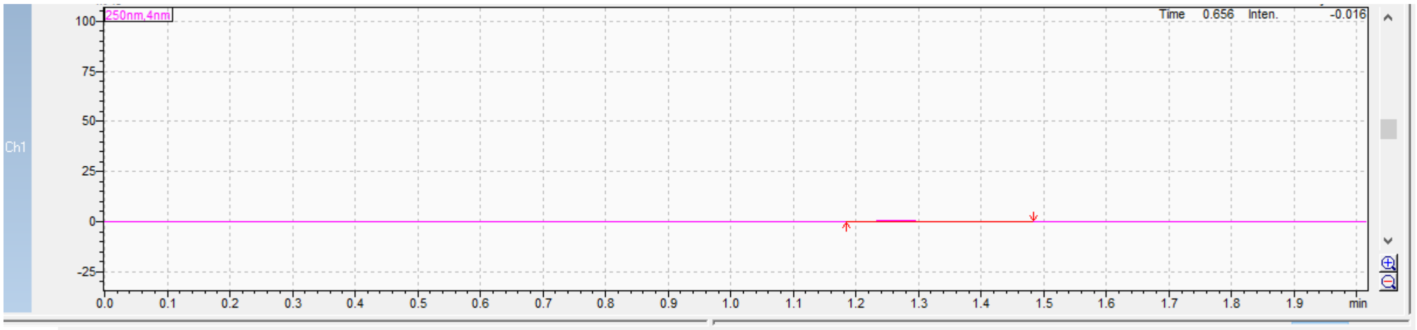

b.

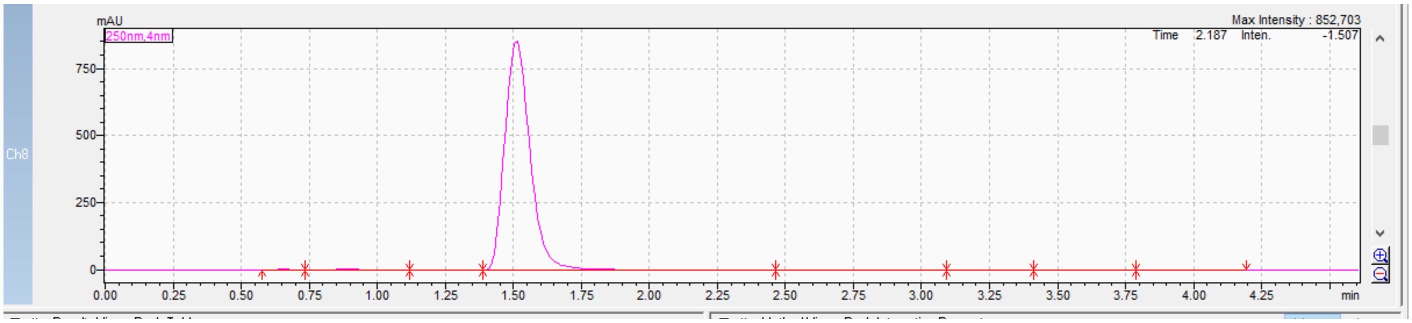

Linearity

This parameter evaluates the relationship between the analyte concentration and the detector response. It establishes the method's ability to produce results directly proportional to the concentration of the Juglone within a specified range. Juglone solutions were created at five different levels, ranging from 2 μg/mL to 200 μg/mL of the working concentration. The data of the area under the curve of the peaks concerning their corresponding concentrations was analyzed using linear regression. The analysis was conducted in triplicate.

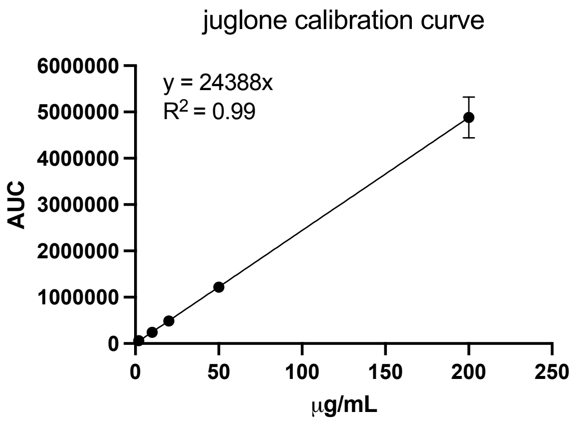

Conc (ug/ml)	AUC 1	AUC 2	AUC 3	Average	SD
**2**	55521	63335	63335	59428	5525
**10**	251053	241273	241273	246163	6916
**20**	477210	478487	478487	477849	903
**50**	1224973	1155152	1155152	1190063	49371
**200**	4786698	4687009	4687009	4736854	70491

Precision

The precision of the assay method was evaluated in terms of repeatability by performing six independent (intra-day) assays of Juglone. This parameter measures the degree of agreement between individual test results and determines the overall precious of the method. Under the same experimental conditions, the intermediate precision of the method was checked by on a different day (inter-day). %RSD not more than 2 was taken as the Limit.

	Repeatability
Sample	Peak area
sample 1	171370
sample 2	178912
sample 3	172334
sample 4	177562
sample 5	174050
sample 6	174945
**Mean**	**174862**
**SD**	**2930**
**%RSD**	**1.67**

Robustness
This parameter measures the ability of the method to remain unaffected by slight variations in analytical parameters such as changes in wavelength, flow rate, temperature, etc. Thus, demonstrating the method's reliability under normal variations observed during routine analysis. The factors chosen for this study were the change in wavelength (+, −3 nm). The appropriate amount of Juglone was weighed and diluted with ethanol. The effect of changed parameters on the analysis of Juglone was evaluated in terms of RT, asymmetry factor, and assay.

		**Retention time (min)**		
**Parameter**		**1**	**2**	**3**
**Change in wavelength**	**+3 nm**	1.517	1.523	1.521
	**−3 nm**	1.478	1.489	1.467

Limit of detection (LOD)
This parameter determines the lowest concentration of Juglone that can be detected reliably but not necessarily quantified with acceptable precision and accuracy. It represents the point where the signal is distinguishable from the background noise. For juglone it was 0.5 μg/mL.

Limit of quantification (LOQ)
This parameter determines the lowest concentration of Juglone that can be detected reliably with acceptable precision and accuracy and can be presented as a specific quantity. For juglone it was 2 μg/mL.

Supplementary information S2

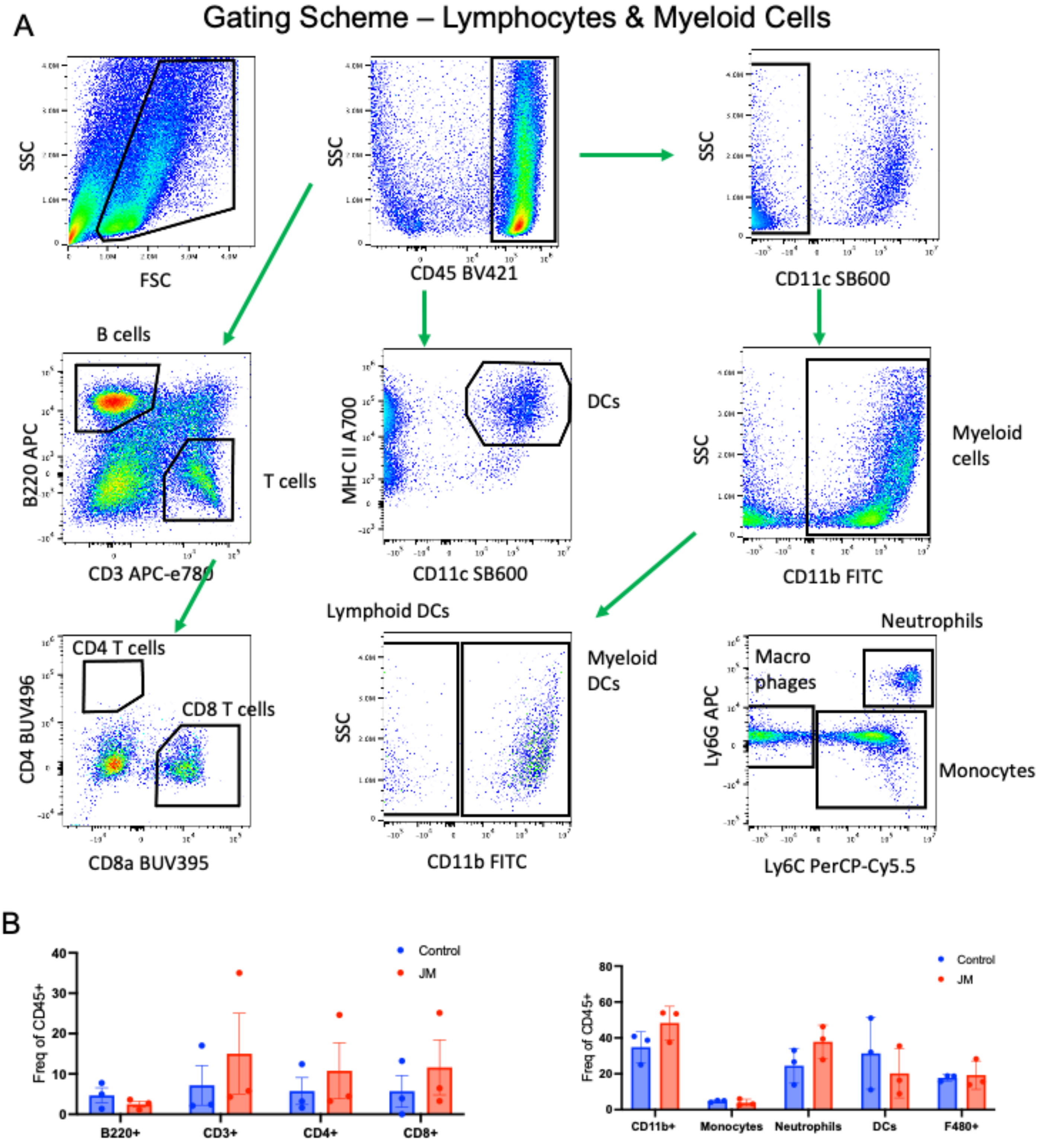

Figure S1. JM treatment modulates immune infiltration, which may help in the antitumor response. (A) Gating strategy of KPC tumors to assess lymphoid and myeloid cell activation by flow cytometric analysis. (B). The proportion of B220+, CD3+, CD4+ CD8+ (lymphoid cells) and Cd11b+, Monocytes, Neutrophils, DCs (dendritic cells), F480+ (myeloid cells) immune cells out of total CD45+ cells as a function of control and JM treated mice assessed by flow cytometric analysis.
